# Is Aesthetic Relational Knowing a Common Factor in Psychotherapy? A Comparison Among Different Models

**DOI:** 10.3390/ejihpe15020016

**Published:** 2025-01-31

**Authors:** Margherita Spagnuolo Lobb, Serena Iacono Isidoro, Claudia Savia Guerrera, Febronia Riggio, Santo Di Nuovo

**Affiliations:** 1 Istituto di Gestalt HCC Italy, 96100 Siracusa, Italy; margherita.spagnuolo@gestalt.it; 2 National Research Council of Italy, Institute for Biomedical Research and Innovation, C/O Via Leanza, Istituto Marino, Mortelle, 98164 Messina, Italy; 3 Department of Educational Sciences, University of Catania, 95131 Catania, Italy; claudia.guerrera@unict.it (C.S.G.); sdinuovo@unict.it (S.D.N.); 4 Department of Cognitive Science, Education and Cultural Studies, University of Messina, 98122 Messina, Italy

**Keywords:** aesthetic relational knowing, Gestalt therapy, psychotherapy models, intuition, embodied awareness, emotional empathy, intuitive resonance

## Abstract

This study explores how aesthetic relational knowing (ARK), as assessed by the ARK-T scale, is used by psychotherapists of different psychotherapeutic models. The ARK-T, a tool based on Gestalt therapy principles, evaluates three core factors of this therapeutic competence: body awareness, affective empathy, and intuitive resonance. A sample of 158 therapists from various approaches, including Gestalt therapy, cognitive–behavioral, systemic–relational, and psychodynamic models, participated in the study. The results show that while body awareness and affective empathy vary in emphasis, depending on the therapeutic approach, intuitive resonance emerges as a shared competence among therapists across orientations. These findings suggest that ARK, particularly the therapist’s capacity to attune to the client’s emotional and relational dynamics, may be a core component of effective therapy. The study highlights the significance of these relational competences in fostering effective therapeutic outcomes across diverse psychotherapeutic frameworks.

## 1. Introduction

Research indicates that some pantheoretical elements found in different effective psychotherapeutic models—called “common factors”—can offer psychotherapists a focusing point to maximize their effectiveness, regardless of their chosen approach (Ahn & Wampold, 2001; Drisko, 2004). According to Wampold and Imel (2015), psychotherapy is effective because of the elements that are common among seemingly disparate models. These common factors found in all psychotherapy models function as a skeleton key to unlock the changes needed for improvement. Development of a therapeutic relationship based on empathy, warmth, and a good working alliance is the most prominent and accepted common factor (Norcross & Wampold, 2011). However, the therapeutic relationship requires more detailed knowledge about the specific variables implied, and thus more research is needed to refine and codify the most useful factors to support training programs (Castonguay, 2000). As a matter of fact, it seems that therapists develop an “informal eclecticism” when their theoretical model proves inadequate (Behan, 2022; Romaioli & Faccio, 2012): there is a natural tendency in clinicians to look for therapeutic factors that they do not perceive as discrepant from what they have learned and include new tools that they find useful, based on practice.

In recent years, research has begun to look at delineating the therapist characteristics that account for a good outcome (Heinonen & Nissen-Lie, 2020), for example, a therapist’s interpersonal characteristics are significant predictors of client benefits (Schöttke et al., 2017). Responsiveness, as Stiles describes it, refers to a therapist’s ability to understand the therapeutic context, perceive nuanced expressions from the client, and intervene in a timely and appropriate manner (Stiles & Horvath, 2017).

This research aimed to explore an insufficiently researched common factor, namely a phenomenological and field-oriented use of the therapist’s feelings in the here and now of the session, to shape their clinical intuition in the therapeutic situation. We hypothesize that therapists learn to intuit their clients using similar relational factors, which we describe here by drawing on a phenomenological, aesthetic, and field perspective. In this context, the field perspective refers to the understanding of therapeutic dynamics as co-constructed processes that emerge within the relational and situational field shared by the therapist and the client. The operationalization of this perspective into concrete clinical thoughts and strategies in the therapists’ mind, beyond their specific approach, could reveal whether these thoughts and strategies represent a common factor in their practice.

We draw on Gestalt therapy principles to describe the experience of the therapist’s responsiveness (Spagnuolo Lobb et al., 2022), focusing on bodily awareness, embodied empathy, and the theory of contact-making between therapist and client (Perls et al., 1951/1994). Specifically, we refer to “aesthetic relational knowing” (ARK) as the therapist’s capacity to stay attuned to their sensorial and emotional experience within the therapeutic encounter. This knowledge is grounded in the aesthetic dimension, which emphasizes the therapist’s ability to perceive the evolving relational process (phenomenological aspect) and to understand the co-created phenomenological field in terms of attachment schemas and habitual relational patterns. ARK is closely tied to what we term “therapeutic intuition”—a broader concept that encompasses the therapist’s ability to integrate these sensorial and emotional cues into timely and appropriate interventions. In this framework, “intuitive competence” captures the procedural skills enabling effective responsiveness, while “intuitive resonance” reflects the attunement to the client’s lived experience. These characteristics of the therapist’s responsiveness describe a crucial procedural aspect of the therapeutic relationship, the intuitive competence of the therapist, and can be measured by a specific instrument, i.e., the Aesthetic Relational Knowledge for Therapists (ARK-T) scale (Spagnuolo Lobb et al., 2024). This scale has been validated to measure the specific “here and now” dynamics of the therapeutic session. The ARK-T scale consists of 21 items which assess three main factors operationalized in the therapeutic context: body awareness, intuitive resonance, and affective empathy.

This study analyzed the responses of 158 therapists from five different psychotherapeutic approaches to the ARK-T scale, aiming to assess the differences in how they use field and aesthetic insight.

The aim was to explore whether epistemologically different approaches enact similar approaches to the patient in clinical practice, or whether they maintain significant differences with respect to the individual factors that make up the construct of ARK.

### 1.1. The Therapist’s Intuition and Relational Experience

Therapeutic intuition is one of the most studied topics in various psychotherapeutic approaches, both in empirical research and clinical studies. Intuition is related to the effectiveness of therapy (Nissen-Lie et al., 2017), to the personal therapy undertaken by the therapist as part of their ongoing professional development (Rønnestad et al., 2016), to the sympathy that certain suffering induces in the therapist (Aponte, 2022), and to factors in the phenomenological/experiential field that is created between therapist and patient (Sarasso et al., 2024). We studied therapeutic intuition according to the latter aspect, drawing on the principles of Gestalt psychotherapy to describe it as a phenomenological and aesthetic event. The therapist’s feelings are considered as an integral aspect of the therapeutic process, as they resonate with and provide insight into the patient’s emotional experience, particularly with regard to attachment patterns and intentionality to resolve suffering. This concept refers to the idea that the therapist’s emotional attunement and resonance actively contribute to therapeutic outcomes.

Several findings support this idea. For instance, Wampold and Imel (2015) have demonstrated that the therapists, as a variable, account for a significant share of client outcomes (about 5%), surpassing other significant variables such as the treatment protocol and client characteristics, which were found to be in the range of 1–2% (Baldwin & Imel, 2013). Following this line of research, efforts have begun to delineate the specific therapist characteristics that contribute to these outcomes (Heinonen & Nissen-Lie, 2020), and there is recent evidence that therapists’ interpersonal characteristics are significant predictors of client outcomes (Schöttke et al., 2017). In particular, more effective therapists are characterized by interpersonal capacities that are professionally cultivated but likely rooted in their personal lives and attachment history—such as empathy, verbal and nonverbal communication skills, and the capacity to form and repair alliances (Heinonen & Nissen-Lie, 2020). Warmth, kindness, and empathy are all characteristics associated with agreeableness, and are essential in the provision of emotional support and conflict resolution (Fletcher & Delgadillo, 2022). These abilities, also observable in trainees and even non-therapists, may reflect the “natural talent” that clinicians bring to their professional work in varying degrees (Orlinsky & Rønnestad, 2005; Nissen-Lie & Orlinsky, 2014); aesthetic sensibilities, rooted in early attachment patterns, may manifest as intuition, wherein attunement and synchronicity play a crucial role in fostering resonance and developing bonds within the therapeutic relationship (Imus & Young, 2023). As a matter of fact, as demonstrated in our previous studies, empathy is not significantly modified by training (Alcaro et al., 2020; Spagnuolo Lobb et al., 2023). There is evidence that therapists’ interpersonal capacities are responsible for the psychotherapy outcome; for instance, the ability to convey empathy, to be affirming, and the capacity to resist counter-aggressive responses to patient hostility or rejection (Nissen-Lie et al., 2013; Andersen et al., 2009; Beutler, 2004; Bohart et al., 2002; Safran et al., 2002; Sandell et al., 2007; Vocisano et al., 2004). We have considered three main qualities of the therapist: their bodily awareness, their capacity to be empathic with the client’s feelings, and their ability to reflect on the previous experience of attachment of the client, using insights gained from their own emotional and relational attunement and resonance within the therapeutic field (Spagnuolo Lobb et al., 2022, 2023, 2024).

The “responsive intuition” described here relates to what is referred to as transference and countertransference in psychodynamic approaches. These processes are regarded as a potential source of beneficial effects for the client, particularly within a scientific landscape that increasingly emphasizes the need to identify in-session change mechanisms driving desirable health outcomes (de Witte et al., 2021). Various studies have shown the strong positive as well as negative impacts of transference work on therapeutic processes and outcomes (Frances & Perry, 1983; Gabbard et al., 1994; Gabbard, 2006; Gelso & Hayes, 2002). Therapists’ focus on countertransference issues may, if not managed, lead to reactions which can adversely affect the quality of therapeutic work (Gelso & Hayes, 2002, 2007). Undoubtedly, therapists’ subjectivity plays an active role in therapy (Aron, 1996; Mitchell, 1993; Renik, 1993), and any clinician should be supported in continuously reflecting on how their feelings can be understood in terms of the phenomenological situation of the client and used for the benefit of the client, favoring a new interplay between them.

As already said, the ARK-T scale (Spagnuolo Lobb et al., 2024) measures therapeutic intuition from a phenomenological, aesthetic, and field perspective. Drawing on the epistemology of Gestalt psychotherapy, this scale explores the therapist’s use of his/her bodily sensations to understand the patient (bodily awareness), the ability to emotionally connect and share in the patient’s emotional experience (affective empathy), and the capacity to integrate sensations that emerge during the therapeutic encounter into the relational patterns that the patient has learned in previous contacts, which now contribute to shape the shared experiential field of the session (intuitive resonance).

Here is a practical illustration of these concepts. A therapist working with a client who has experienced childhood neglect may notice a tightness in their own chest (bodily awareness) during a session. This sensation allows the therapist to remain grounded while empathizing with the client’s sadness and feelings of abandonment (affective empathy). By reflecting on how the tightness in their chest connects to the client’s relational patterns, the therapist wonders how this feeling can “match” with the client’s sadness and sense of abandonment, facilitating a deeper understanding of the phenomenological field they both experience (intuitive resonance). The tightness in the chest of the therapist and the sadness of the client are linked the one to the other and create an experiential field that will be transformed by a tailored therapeutic intervention. By engaging with the client’s emotional experience in a relational and processual way, the therapist fosters a deeper connection and facilitates meaningful therapeutic work, demonstrating the relevance of the ARK framework in clinical practice.

This instrument of perception therefore draws on the therapist’s sensory capacity and also on his or her ability to place sensations in a field perspective. We understand ARK as a key factor in therapeutic competence, and the ARK-T scale offers a validated tool to measure this intuitive, embodied relational knowing in therapeutic practice.

### 1.2. Hypotheses of This Study

The study was guided by several hypotheses regarding the therapists’ aesthetic intuition and specific factors of the ARK-T scale in different models.

The first hypothesis (H1), relevant to therapist training, explored whether therapists’ aesthetic intuition differs significantly, based on their personal characteristics, e.g., years of experience and gender.

The second hypothesis (H2) suggested that the above-described form of “responsive intuition”, although theoretically rooted in Gestalt therapy, is likely used by therapists from other approaches.

The third hypothesis (H3) posited those specific factors of the ARK-T scale—body awareness, affective empathy, and intuitive resonance—are distributed differently across therapeutic approaches.

The fourth hypothesis (H4) examined whether there is a significant difference in aesthetic intuition between therapists who work predominantly online and those who work in person.

## 2. Materials and Methods

### 2.1. Sample

The inclusion criteria for the study required that participants be psychotherapists certified and active in accordance with Italian law (No. 56/1989), which limits training and professional activity in psychotherapy to specialized psychologists and physicians. The sample included 158 therapists (44 male, 114 female) between the ages of 25 and 80 years, practicing in various regions of Italy. The gender disparity in the sample reflects the broader reality of the field, supported by data indicating a strong female predominance in the psychological professions, with about 80 percent of clinical psychologists being women (Johnson et al., 2020; Yang, 2024).

All participants were experienced practitioners who specialized in different psychotherapeutic approaches and represented a range of theoretical, methodological, cultural, and professional backgrounds. The sample included 31 therapists belonging to psychoanalytical and psychodynamic schools; 36 therapists specializing in cognitive, cognitive–behavioral, and cognitive–evolutionary approaches; 30 systemic–relational therapists; 30 Gestalt therapists; and 31 neo-functional therapists^1^.

The characteristics of the sample and composition of the subgroups are summarized in Table 1.

Significant gender differences are shown across the various psychotherapeutic approaches (χ^2^ = 19.66, df = 4, *p* < 0.001). Indeed, the predominance of females is found in all approaches except for the psychodynamic/psychoanalytic group, in which the number of male therapists is higher than females. However, no significant differences are found regarding ages (χ^2^ = 22.80, df = 8, *p* = 0.30) or years of experience (χ^2^ = 24.31, df = 20, *p* = 0.23). The substantial homogeneity of the groups in these variables, crucial for the aims of the research, ensures the reliability of the comparisons for subsequent analyses.

### 2.2. Tools and Procedures

Participants were invited via e-mail by the presidents of the psychotherapy associations involved, or by colleagues. After giving informed consent, they received an invitation to complete a questionnaire and the ARK-T scale. After they completed the scale items, an open-ended question was administered about the subjective definition of “therapist’s intuition”.

Data were collected anonymously via a selected link between September 2023 and January 2024.

#### 2.2.1. Socio-Demographic Questionnaire

A questionnaire with socio-demographic variables was used. Gender, age, region of residence, years of experience, psychotherapeutic approach, and modality (online or in person) of conducting psychotherapy sessions were investigated.

#### 2.2.2. Aesthetic Relational Knowledge for Therapists (ARK-T) Scale

The ARK-T scale (Spagnuolo Lobb et al., 2024) measures the aesthetic intuition of the therapist in the here and now of the session. It assesses the therapist’s ability to integrate their bodily awareness, empathic understanding, and resonance into the therapeutic field. The construct of ARK (Spagnuolo Lobb et al., 2022; Alcaro et al., 2020) was initially described as composed of three aspects: bodily awareness, empathy, and resonance. The validation study of the scale (Spagnuolo Lobb et al., 2024) finally presented a description of the factors composing the scale that were named bodily awareness, affective empathy, and intuitive resonance. The scale is composed of 21 items, representing the three factors: 8 for body awareness, 5 for affective empathy, and 8 for intuitive resonance. The scale showed good reliability (α = 0.841).

After the rotation of the scores’ direction for the reversed items, each factor allows the sum of the corresponding subscale score to be used for research purposes.

### 2.3. Statistical Analysis

Descriptive and parametric statistics were used to analyze the sample data. Independent sample *t*-tests and analysis of variance (ANOVA) were used to investigate differences related to socio-demographic variables. The *t*-test was also used to examine ARK-T scores for some target variables (gender and predominant work modality, online or in person). ANOVAs were performed to compare the mean scores of ARK-T by psychotherapeutic approach and years of experience.

Systat 13 (SYSTAT Software, Inc., Chicago, IL, USA) was used for the analyses.

## 3. Results

### 3.1. H1: Influence of Therapist Characteristics (Years of Experience and Gender)

As a preliminary analysis, an independent sample *t*-test was performed to examine the differences in the mean ARK-T scores between male and female participants. No significant difference was found in the mean total score of the scale (t = 0.07, df = 156, *p* = 0.95). Additionally, the *t*-test was applied to investigate differences in the mean scores of the individual component factors of the scale. In this regard, while no significant differences in mean scores were observed between genders for the body awareness and affective empathy factors, the difference in mean scores for the intuitive resonance factor was statistically significant, with higher scores for males (t = 2.79, df = 156, *p* = 0.01). These findings suggest that gender influences intuitive resonance, while the other factors remain unaffected by gender (Table 2).

Analysis of variance for the ARK-T total score by years of experience revealed slightly higher scores among therapists with 20 to 40 years of experience. However, the difference was not statistically significant. In general, experience does not seem to affect competence regarding aesthetic relational knowing.

### 3.2. H2: Use of Responsive Intuition Across Approaches

Analysis of variance for the ARK-T total score and specific factors by therapeutic model showed highly significant differences (Table 3).

The ARK-T total score was higher for the Gestalt therapy and Functional therapy models compared with other therapeutic models, with intermediate scores for the systemic–relational model and the lowest scores for the psychoanalytic model (F = 13.3, df = 4, 153; *p* < 0.001).

See also Figure 1.

Given the significance of the differences in the ARK-T total score, a detailed analysis was performed for individual factors to identify the components contributing most to the observed overall differences among the treatment models.

### 3.3. H3: Distribution of ARK-T Factors Across Therapeutic Approaches

By examining individual factors, we can better understand the dimensions of the ARK-T scale that may be most sensitive to the therapeutic approaches, offering more focused insights into how different models shape therapists’ awareness and empathy. This approach allows for a more nuanced interpretation of the results, highlighting areas where the therapeutic model may have a greater impact.

**Figure 2:**
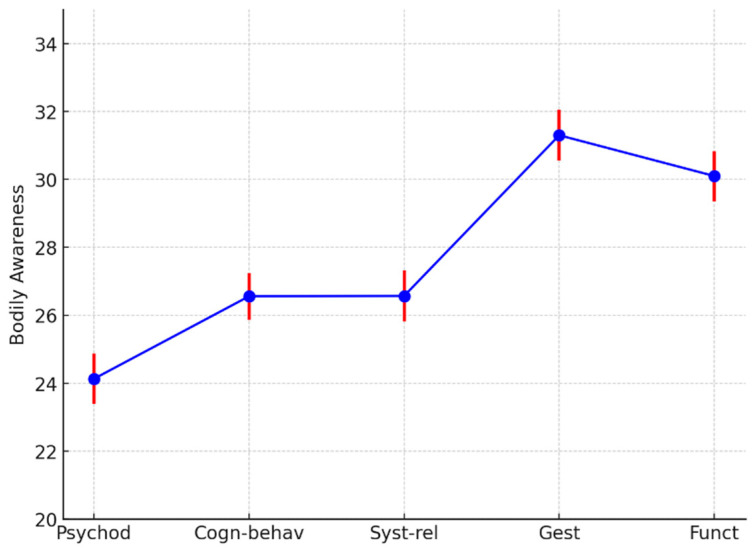
Body awareness least squares means by psychotherapy models. Psychod, psychoanalytic and psychodynamic therapists; Cogn-behav, cognitive, cognitive–behavioral, and cognitive–evolutionary approaches; Syst-Rel, systemic–relational therapists; Gest, Gestalt therapists; Func, functional therapists. The different colors are used to represent distinct elements of the data: blue represents the mean values of the ARK-T Total score for each category (Psychod, Cogn-behav, Syst-rel, Gest, Funct); red represents the error bars for the corresponding mean values. The combination of these colors helps visually distinguish the data points (blue) from the variability in the measurements (red).

For the “body awareness” factor, Gestalt and functional therapists scored higher (F = 15.5, df = 4, 153; *p* < 0.001), showing the same trend as the ARK-T total score (Table 3, Figure 2).

**Figure 3:**
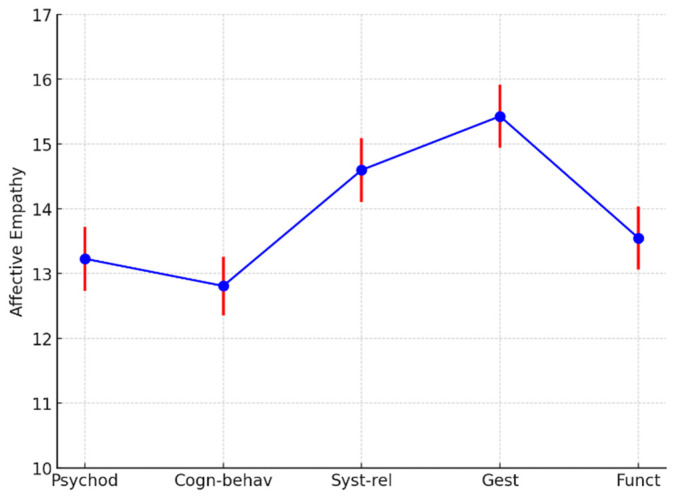
Affective empathy least squares means by psychotherapy models. Psychod, psychoanalytic and psychodynamic therapists; Cogn-behav, cognitive, cognitive–behavioral, and cognitive–evolutionary approaches; Syst-Rel, systemic–relational therapists; Gest, Gestalt therapists; Func, functional therapists. The different colors are used to represent distinct elements of the data: blue represents the mean values of the ARK-T Total score for each category (Psychod, Cogn-behav, Syst-rel, Gest, Funct); red represents the error bars for the corresponding mean values. The combination of these colors helps visually distinguish the data points (blue) from the variability in the measurements (red).

For the “affective empathy” factor, statistically significant differences were also observed (F = 5.0, df = 4, 153; *p* < 0.001), with higher scores for Gestalt and systemic–relational therapists, and the lowest scores for the cognitive–behavioral model (Figure 3).

**Figure 4:**
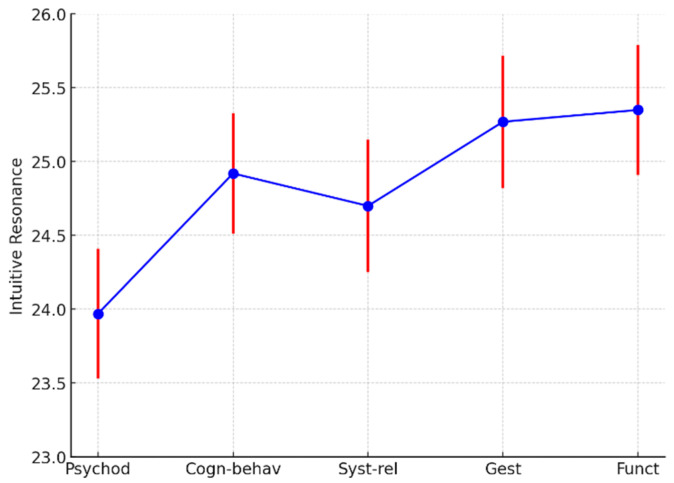
Intuitive resonance least squares means by psychotherapy models. Psychod, psychoanalytic and psychodynamic therapists; Cogn-behav, cognitive, cognitive–behavioral, and cognitive–evolutionary approaches; Syst-Rel, systemic–relational therapists; Gest, Gestalt therapists; Func, functional therapists. The different colors are used to represent distinct elements of the data: blue represents the mean values of the ARK-T Total score for each category (Psychod, Cogn-behav, Syst-rel, Gest, Funct); red represents the error bars for the corresponding mean values. The combination of these colors helps visually distinguish the data points (blue) from the variability in the measurements (red).

For the “intuitive resonance” factor, no significant differences were found among the groups (F = 1.60, df = 4, 153; *p* = 0.18) (Figure 4).

These results suggest that while Gestalt and functional therapists demonstrate greater body awareness, and Gestalt and systemic–relational therapists show higher affective empathy, intuitive resonance appears to be consistent across therapeutic models.

### 3.4. H4: Influence of Online Therapy on ARK-T Scores

Finally, we analyzed the differences in ARK-T scores in relation to the use of online therapies.

In the overall sample, therapists who use online interventions for more than half of their sessions did not differ significantly in mean ARK-T scores from those who use online interventions less frequently: group < 50% (n = 71), ARK-T mean = 66.57, st. dev. = 7.63; group ≥ 50%, (n = 21), ARK-T mean = 66.14, st. dev. = 7.23; t = 0.23, df = 89, *p* = 0.82.

This result suggests that therapists’ aesthetic intuition is unaffected by whether they work predominantly online or in person.

## 4. Discussion

### 4.1. H1: Gender and Years of Clinical Experience

The study examined whether therapists’ aesthetic intuition, as measured by the ARK-T scale, differs according to gender and years of experience. No significant difference was found related to clinical experience, with only a non-significant trend observed in therapists with 20 to 40 years of experience. This raises questions about whether intuitive capacity—here referring specifically to the ability to attune to and resonate with the client’s experience, as operationalized by the concept of intuitive resonance—is primarily linked to basic training or more strongly influenced by accumulated clinical experience. This is consistent with other findings indicating that factors such as years of practice do not significantly influence therapists’ performance (Wolfer et al., 2022) and may not explain variance components in therapists’ effectiveness (Castonguay, 2000; Behan, 2022; Romaioli & Faccio, 2012; Heinonen & Nissen-Lie, 2020; Schöttke et al., 2017; Stiles & Horvath, 2017; Spagnuolo Lobb et al., 2022, 2023, 2024; Perls et al., 1951/1994; Nissen-Lie et al., 2013, 2017; Rønnestad et al., 2016; Aponte, 2022; Sarasso et al., 2024; Baldwin & Imel, 2013; Fletcher & Delgadillo, 2022; Orlinsky & Rønnestad, 2005; Nissen-Lie & Orlinsky, 2014; Imus & Young, 2023; Alcaro et al., 2020; Andersen et al., 2009; Beutler, 2004; Bohart et al., 2002; Safran et al., 2002; Sandell et al., 2007; Vocisano et al., 2004; de Witte et al., 2021; Frances & Perry, 1983; Gabbard et al., 1994; Gabbard, 2006; Gelso & Hayes, 2002, 2007; Aron, 1996; Mitchell, 1993; Renik, 1993; Johnson et al., 2020; Yang, 2024; Wolfer et al., 2022; Brown et al., 2005; Okiishi et al., 2003; Goldberg et al., 2016) or even negative effects (Lingiardi et al., 2018). Future longitudinal research could clarify this relationship.

Regarding gender, no significant difference was observed in the overall use of the ARK-T. However, males scored significantly higher on the intuitive resonance factor. This result may stem from multiple factors, including social perception and expectations associated with gender roles. In some contexts, men might feel more motivated to develop relational skills, such as intuitive resonance, to counterbalance stereotypes that perceive them as less empathic compared with women. This could lead to a conscious effort to refine these abilities, particularly in therapeutic settings, resulting in higher scores. Additionally, men may exhibit greater confidence in attributing such abilities to themselves, potentially leading to a perceptual bias in self-reporting these abilities. The sample composition may also play a role, as males were more represented in psychoanalytic methods, which frequently utilize countertransference as a central aspect of their psychotherapeutic interventions.

### 4.2. H2: Differences Across Therapeutic Approaches

In this study, we hypothesize that therapists intuit their clients using similar relational factors, beyond their specific approach. These factors are described using categories derived from Gestalt therapy, which is a phenomenological perspective, emphasizing the therapist’s attunement to the lived experience of the therapeutic encounter as it unfolds in the present moment; an aesthetic perspective, focusing on the sensorial and emotional resonance emerging within the relational process; and a field perspective, considering the co-created relational and situational context shared by the therapist and client.

These perspectives offer a conceptual framework for understanding therapists’ responsiveness. The ARK concept translates therapists’ responsiveness into measurable constructs applicable to the therapeutic session. The ARK-T scale, a validated tool based on phenomenological, aesthetic, and field perspectives, operationalizes this concept into concrete clinical thoughts and strategies employed by therapists. Findings from administering the ARK-T scale to psychotherapists from five different approaches suggest that such thoughts and strategies represent common factors in their practice, but only for certain aspects.

With regards to the second hypothesis—i.e., that the ARK is likely used by therapists from different approaches—we have found that psychotherapists who most extensively use this competence are Gestalt therapists and neo-functionalists. Systemic–relational therapists use this competence to an intermediate extent, while psychoanalysts use it less. These results suggest that ARK is employed differently, depending on the therapeutic model, supporting a dialogue among therapeutic approaches and training programs.

### 4.3. H3: Specific Factors of the ARK-T Scale

The study further explored whether specific factors of the ARK-T scale—body awareness, affective empathy, and intuitive resonance—are distributed differently across therapeutic approaches. The findings largely confirmed this hypothesis. As expected, body awareness was most prominent among Gestalt therapy and neo-functional therapists, reflecting the body-centered approaches focus of these approaches; affective empathy was more pronounced among Gestalt and systemic–relational therapists, whose practices emphasize emotional attunement and attachment processes; intuitive resonance, however, showed no significant differences across therapeutic models. This suggests that the capacity to situate the sensations that emerge in the therapeutic situation with the patient within the relational patterns learned in previous contacts, and that now contribute to forming the experiential field experienced by both in the session, is broadly shared among therapists, beyond the method.

These findings support the idea that different therapeutic models emphasize distinct relational and empathic skills, with intuitive resonance representing a process deeply rooted in the therapist’s capacity to attune to the co-created relational field and respond authentically to the client’s experience, emphasizing its immediacy and intersubjective quality.

### 4.4. H4: Online vs. In-Person Therapy

The study also investigated whether therapists’ aesthetic intuition differs between therapists who work predominantly online and those who work in person. The results indicated no significant differences between these groups. This finding suggests that the mode of intervention—online versus in person—does not significantly influence the therapist’s competence in relational knowing as measured by the ARK-T scale.

### 4.5. Limits of the Study and Future Research

Although the psychotherapists who have participated in this study clearly identified with a specific method, some relevant differences inside each identified method have not been considered. For instance, there are differences within the category “psychoanalysis and psychodynamic” regarding the relational aspects of the therapeutic relationship. The same can be said regarding the cognitive–behavioral approach. More detailed analysis of single approaches could be taken into account in future studies on ARK variables.

Moreover, it would be useful to compare the ARK-T scores with specific training aspects, in order to enquire about the presence of this competence as an effect of training or, prevalently, of clinical experience.

Finally, we hope that further research will study the connection between psychotherapy’s effectiveness and intuitive competence, measured with the ARK-T scale, to explore how the three factors that compose the aesthetic relational knowing contribute to facilitating the changes needed for therapeutic improvement. By “changes”, we refer to the therapeutic shifts in relational patterns, emotional regulation, and self-awareness that clients experience as part of the psychotherapeutic process. This type of study would also help better delineate ARK as one of the therapist’s interpersonal characteristics associated with positive outcomes, while ARK-T serves as the tool to measure these characteristics. Additionally, it is important to emphasize that all therapist training programs aim to enhance the competencies and responsiveness of therapists, regardless of their specific approach. Exploring how ARK-related skills are supported within training contexts could provide valuable insights for integrating these competencies into diverse training programs. This would represent an important next step in understanding and applying the ARK model across different therapeutic paradigms.

## 5. Conclusions

The ARK-T scale (Spagnuolo Lobb et al., 2024) assesses a crucial procedural aspect of the therapeutic relationship, namely the aesthetic intuitive competence of the therapist, which is the capacity to understand the therapeutic context, detect nuanced expressions from the client, and intervene in a timely and appropriate manner.

In this study, we have found that the total ARK-T score appears to be influenced by the model of therapy in which therapists are trained, rather than by the environmental setting (online or in person), years of practice, or gender. However, a significant difference based on gender was observed specifically in the intuitive resonance factor, with higher scores reported for males. This raises the question of whether training programs shape relational competencies or simply refine inherent traits of the therapists.

Further research is needed to explore how relational skills (such as intuitive resonance) are supported and cultivated across training in various therapeutic approaches. To examine how therapist training impacts the development of such competencies would provide a deeper understanding of the interplay between training and personal predispositions, especially considering the ARK model.

The therapeutic model—and therefore psychotherapy training and experience—appears to influence two specific factors of this competence, namely body awareness and affective empathy, while a third factor, intuitive resonance, seems to be a common competence among therapists.

This result seems to be partially in line with previous studies that highlighted how the general interpersonal therapeutic skills of therapists (including empathy, warmth, clear communication, and appreciation) represent significant and consistent qualities that therapists can positively rely on in the patient–therapist interaction (Heinonen & Nissen-Lie, 2020; Wampold, 2012). Together with Wampold (Lingiardi et al., 2018) we can say that, although there are many forms of psychotherapy, each distinctive in its own way, from the origins of psychotherapy, it has been suggested this profession is effective through factors that are common to all therapies. In this article, we suggest that one common factor that is at the core of psychotherapeutic relation is intuitive resonance: the capacity to understand the therapist’s feelings as part of an experiential field co-created with the client and locate them in the attachment expectations and resilience available to the client. Further studies are needed to evaluate the efficacy of intuitive resonance in the context of specific clinical conditions. Such efforts could contribute to the creation of a “shared ontology” across different psychotherapy schools by identifying “wide-spectrum” strategies for change grounded in a meta-theoretical framework (De Felice et al., 2019).

The other two aspects of the therapeutic aesthetic intuition—body awareness (being aware of one’s own body) and affective empathy (being empathic with affective aspects of the client’s relational patterns and attachment habits)—are related to the specific model in which the therapists are trained.

The concept of the ARK of the therapist offers a framework for psychotherapists to focus and maximize their intuition during the session. In particular, one aspect of this competence, intuitive resonance, emerged as a shared quality across a wide range of approaches, demonstrating consistency among different therapeutic models. This finding aligns with research suggesting that some relational competences transcend specific modalities and function as common factors in psychotherapy. The ARK model, by operationalizing these elements, provides therapists with a structured way to refine their intuitive competences, thereby supporting the therapeutic process.

This result is significant for understanding not only how epistemological differences among psychotherapy models manifest in clinical practice but also for exploring the measurable components of therapists’ responsiveness. Although each psychotherapy model has its own unique theoretical foundations and techniques, some relational factors, such as empathy and resonance, are shared across approaches and contribute to therapeutic efficacy by transcending any specific method.

The ARK-T, as a structured tool for assessing intuitive and relational competences, offers an innovative way to measure previously difficult-to-capture aspects of therapy. The ARK model allows for a reliable comparison of therapeutic modalities on specific aspects such as body awareness, affective empathy, and intuitive resonance. By operationalizing and coding these components into measurable constructs, ARK helps bridge the theoretical gap between models, offering a unified perspective on the core competencies that underlie therapeutic success. Furthermore, these findings open new avenues for reflection on the role of training programs and therapeutic frameworks in developing these competences. Targeted training programs can leverage ARK’s insights to cultivate these essential relational skills, which are valuable in all therapeutic approaches. Further research is needed to investigate how these differences arise—whether they stem primarily from training, inherent therapist characteristics, or specific theoretical orientations—and to examine how the ARK model can inform and support therapist education across modalities.

## Figures and Tables

**Figure 1:**
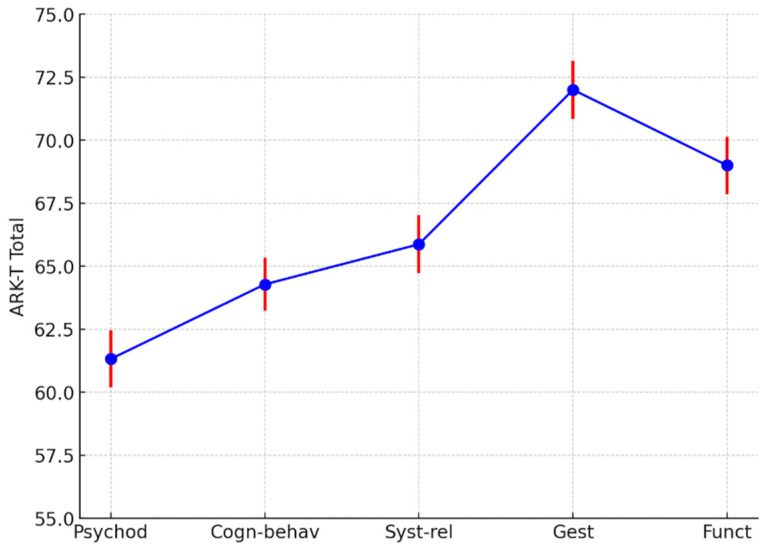
ARK-T total least squares means by psychotherapy models. Psychod, psychoanalytic and psychodynamic therapists; Cogn-behav, cognitive, cognitive–behavioral, and cognitive–evolutionary approaches; Syst-Rel, systemic–relational therapists; Gest, Gestalt therapists; Func, functional therapists. The different colors are used to represent distinct elements of the data: blue represents the mean values of the ARK-T Total score for each category (Psychod, Cogn-behav, Syst-rel, Gest, Funct); red represents the error bars for the corresponding mean values. The combination of these colors helps visually distinguish the data points (blue) from the variability in the measurements (red).

**Table 1:** Sample characteristics and subgroups.

	Tot	Psychod	Cogn-Behav	Syst-Rel	Gest	Func
N	158	31	36	30	30	31
Gender						
Male	44	18	10	4	7	5
Female	114	13	26	26	23	26
Age						
25–35	26	3	6	8	5	4
36–45	61	10	13	10	11	17
46–55	43	6	13	7	10	8
56–65	14	5	2	4	2	1
66–75	9	4	2	1	2	0
76–85	5	3	1	0	0	1
Years of experience						
<5 yrs	46	3	9	12	7	15
5–10 yrs	40	8	12	4	9	7
10–20 yrs	32	6	6	7	7	6
20–30 yrs	22	6	5	5	4	2
30–40 yrs	7	3	1	1	1	1
>40 yrs	11	5	3	1	2	0

Groups: Tot, total sample; Psychod, psychoanalytic and psychodynamic therapists; Cogn-Behav, cognitive, cognitive–behavioral, and cognitive–evolutionary approaches; Syst-Rel, systemic–relational therapists; Gest, Gestalt therapists; Func, functional therapists.

**Table 2:** Differences in ARK-T mean scores (total and factors) by gender.

ARK-T	Gender	Mean (SD)	*t*-Test		
			t	df	*p*-Value
Total	Male	66.45 (7.28)	0.07	156	0.95
	Female	66.37 (7.25)			
Body awareness	Male	27.34 (5.15)	0.54		0.59
	Female	27.81 (4.71)			
Affective empathy	Male	13.41 (2.53)	1.28		0.20
	Female	14.05 (2.95)			
Intuitive resonance	Male	25.70 (2.73)	2.79		0.01
	Female	24.51 (2.33)			

**Table 3:** ARK-T total and factors’ mean scores by psychotherapeutic model.

ARK-T	Model	N	Mean	St. Error	df	F-Ratio	*p*-Value
Total	Psychod	31	61.32	1.13	4 153	13.3	<0.001
	Cogn-Behav	36	64.28	1.05			
	Syst-Rel	30	65.87	1.15			
	Gest	30	72.00	1.15			
	Func	31	69.00	1.13			
Body awareness	Psychod	31	24.13	0.74		15.5	<0.001
	Cogn-Behav	36	26.56	0.69			
	Syst-Rel	30	26.57	0.75			
	Gest	30	31.30	0.75			
	Func	31	30.10	0.74			
Affective empathy	Psychod	31	13.23	0.49		5.00	<0.001
	Cogn-Behav	36	12.81	0.45			
	Syst-Rel	30	14.60	0.49			
	Gest	30	15.43	0.49			
	Func	31	13.55	0.49			
Intuitive resonance	Psychod	31	23.97	0.44		1.60	0.18
	Cogn-Behav	36	24.92	0.41			
	Syst-Rel	30	24.70	0.45			
	Gest	30	25.27	0.45			
	Func	31	25.35	0.44			

Groups: Tot, Total sample; Psychod, psychoanalytic and psychodynamic therapists; Cogn-Behav, cognitive, cognitive–behavioral, and cognitive–evolutionary approaches; Syst-Rel, systemic–relational therapists; Gest, Gestalt therapists; Func, functional therapists.

## Data Availability

The original contributions presented in the study are included in the article; further inquiries can be directed to the corresponding author/s.

## Note

1 Neo-functional therapy focuses on improving overall well-being by working on four key areas: body, emotions, thoughts, and identity. It emphasizes the recovery of fundamental experiences, such as breathing, posture, and emotional expression, to promote psychological balance and holistic harmony.
